# Association of Children’s Physical Activity and Screen Time With Mental Health During the COVID-19 Pandemic

**DOI:** 10.1001/jamanetworkopen.2021.27892

**Published:** 2021-10-01

**Authors:** Pooja S. Tandon, Chuan Zhou, Ashleigh M. Johnson, Erin Schoenfelder Gonzalez, Emily Kroshus

**Affiliations:** 1 Center for Child Health, Behavior, and Development, Seattle Children’s Research Institute, Seattle, Washington; 2 Department of Pediatrics, University of Washington, Seattle; 3 Department of Psychiatry and Behavioral Sciences, University of Washington, Seattle

## Abstract

**Importance:**

Children’s physical activity and screen time are likely suboptimal during the COVID-19 pandemic, which may influence their current and future mental health.

**Objective:**

To describe the association of physical activity and screen time with mental health among US children during the pandemic.

**Design, Setting, and Participants:**

This cross-sectional survey was conducted from October 22 to November 2, 2020, among 547 parents of children aged 6 to 10 years and 535 parent-child dyads with children and adolescents (hereinafter referred to as children) aged 11 to 17 years and matched down to 500 children per cohort using US Census–based sampling frames. Children aged 11 to 17 years self-reported physical activity, screen time, and mental health, and their parents reported other measures. Parents of children aged 6 to 10 years reported all measures. All 1000 cases were further weighted to a sampling frame corresponding to US parents with children aged 6 to 17 years using propensity scores.

**Exposures:**

Child physical activity, screen time, COVID-19 stressors, and demographics.

**Main Outcomes and Measures:**

Mental health using the Strengths and Difficulties Questionnaire for total difficulties and externalizing and internalizing symptoms.

**Results:**

Among the 1000 children included in the analysis (mean [SD] age, 10.8 [3.5] years; 517 [52.6%] boys; 293 [31.6%] American Indian/Alaska Native, Asian, or Black individuals or individuals of other race; and 233 [27.8%] Hispanic/Latino individuals), 195 (20.9%) reported at least 60 minutes of physical activity every day. Children reported a mean (SD) of 3.9 (2.2) d/wk with at least 60 minutes of physical activity and 4.4 (2.5) h/d of recreational screen time. COVID-19 stressors were significantly associated with higher total difficulties for both younger (β coefficient, 0.6; 95% CI, 0.3-0.9) and older (β coefficient, 0.4; 95% CI, 0.0-0.7) groups. After accounting for COVID-19 stressors, engaging in 7 d/wk (vs 0) of physical activity was associated with fewer externalizing symptoms in younger children (β coefficient, −2.0; 95% CI, −3.4 to −0.6). For older children, engaging in 1 to 6 and 7 d/wk (vs 0) of physical activity was associated with lower total difficulties (β coefficients, −3.5 [95% CI, −5.3 to −1.8] and −3.6 [95% CI, −5.8 to −1.4], respectively), fewer externalizing symptoms (β coefficients,  −1.5 [95% CI, −2.5 to −0.4] and −1.3 [95% CI, −2.6 to 0], respectively), and fewer internalizing symptoms (β coefficients, −2.1 [95% CI, −3.0 to −1.1] and −2.3 [95% CI, −3.5 to −1.1], respectively). More screen time was correlated with higher total difficulties among younger (β coefficient, 0.3; 95% CI, 0.1-0.5) and older (β coefficient, 0.4; 95% CI, 0.2-0.6) children. There were no significant differences by sex.

**Conclusions and Relevance:**

In this cross-sectional survey study, more physical activity and less screen time were associated with better mental health for children, accounting for pandemic stressors. Children engaged in suboptimal amounts of physical activity and screen time, making this a potentially important target for intervention.

## Key Points

**Question:**

What is the association of children’s physical activity and screen time with mental health during the COVID-19 pandemic?

**Findings:**

In this cross-sectional national survey that included 1000 school-aged children in the US, children who engaged in more physical activity and less screen time had better mental health outcomes as measured by the Strengths and Difficulties Questionnaire.

**Meaning:**

These findings suggest that physical activity and screen time may be targets for promoting children’s mental health during and after the COVID-19 pandemic.

## Teaser

This cross-sectional survey study assesses the association of physical activity and screen time with the mental health of school-aged children in the US during the COVID-19 pandemic.

## Introduction

The COVID-19 pandemic has led to serious disruptions in the lives of children, including restrictions on school and other activities, and numerous other stressors that may continue to pose challenges to their health and well-being. Emerging evidence raises concern that such stressors may have negative short- and long-term mental health consequences, including psychological distress and behavioral problems.^1,2,3^ Identifying potentially modifiable factors that are positively associated with youth mental health can provide the foundation for approaches to support children as they continue to experience pandemic-related stressors. Physical activity and screen time are 2 key health behaviors that are likely affected by the COVID-19 pandemic and may influence differential mental health outcomes.

Physical activity is associated with numerous physical and mental health benefits across the lifespan.^4^ Current evidence supports the notion that physical activity in children is associated with protection against internalizing^5^ (eg, depression, anxiety) and externalizing^6^ (eg, impulse control, aggressiveness) symptoms and can improve positive psychosocial functioning.^7^ The COVID-19 pandemic has magnified the importance of research focusing on the mental health benefits of physical activity.^8^ Two studies from China found that children who engaged in more physical activity during the pandemic reported better behavioral and mental health.^2,3^ Experts are calling for more active promotion of physical activity to be a key recommendation to support population health during and beyond the COVID-19 pandemic.^8,9^

Despite the potential benefits of physical activity on child well-being, emergent data suggest that during this pandemic, access to health-promoting physical activity has been largely disrupted, whereas use of screen-based media for education and recreation has increased.^10,11,12^ Studies from before the pandemic indicate that only 24% of US children and adolescents aged 6 to 17 years met recommendations for 60 minutes per day of physical activity.^4^ Data from the 2017 National Youth Risk Behavior Survey showed that 26.1% of high school students had been physically active for a total of at least 60 min/d on all 7 days before the survey.^13^ Further, physical activity levels have been shown to be negatively associated with age (lower for older children),^14^ positively associated with parental educational attainment,^15,16,17^ and lower among girls (vs boys) and racial and ethnic minority groups.^14,18^ Before the pandemic, school-aged children in the US also engaged in excessive amounts of screen time, with estimates ranging from 4 to more than 7 h/d.^19,20^ With most schools in the US opting for remote schooling at some point during the pandemic, children are likely spending more time sedentary and in front of screens than ever before. While recognizing the benefits of some types of screen use, excessive screen time has been associated with lower psychological well-being.^21,22^

Several studies in adults during the pandemic have found consistent associations between lower physical activity and poorer mental health and between higher daily screen time and poorer mental health,^10,23,24^ but association this has not been well studied in US children. Researchers have drawn on prepandemic studies^25,26,27,28,29,30,31,32^ to emphasize the potential mental health consequences of the pandemic and called for research and augmented mental health services. However, few studies have reported on the mental health of children during the pandemic and none, to our knowledge, have specifically examined the association of physical activity and screen time with mental health in nationally representative US samples.^33,34^ The aims of this study are to describe the association of physical activity and screen time with the mental health of school-aged children in the US during the COVID-19 pandemic. We also sought to understand how these health behaviors were affected by children’s exposure to pandemic-related stressors.

## Methods

### Participants

A market research company (YouGov) conducted an online, opt-in, cross-sectional survey from October 22 to November 2, 2020, and generated a US nationally representative sample of 500 parent-child dyads with children and adolescents (hereinafter referred to as children) aged 11 to 17 years and 500 parents of children aged 6 to 10 years. YouGov surveyed 547 parents of children aged 6 to 10 years and 535 parent-child dyads with children aged 11 to 17 years. Respondents were then matched down to samples of 500 in each cohort according to US Census–based sampling frames by age, race, and educational level for children aged 6 to 10 years and by sex, age, race, and educational level for children aged 11 to 17 years. Both frames were constructed by stratified probability sampling from the full 2017 American Community Survey 1-year sample, with selection within strata by weighted sampling with replacements (using the person weights on the public use file). All parents of children aged 11 to 17 years also recruited one of their children aged 11 to 17 years for a survey. All 1000 cases were further weighted to a sampling frame corresponding to US parents with children aged 6 to 17 years. Weighting was performed using propensity scores. The matched cases and the frame were combined, and a logistic regression model was estimated to construct propensity of inclusion in the frame vs the matched cases. The logistic regression included age, sex, race and ethnicity, years of education, and region. Sampling weights, which were inversely proportional to the propensity of being in the matched cases, were used in statistical analysis to reduce bias. Details for YouGov’s sampling matching procedure can be found in Rivers.^35^

This study followed the American Association for Public Opinion Research (AAPOR) best practices for survey research and the Strengthening the Reporting of Observational Studies in Epidemiology (STROBE) reporting guideline for cross-sectional studies. Research procedures were approved by the institutional review board of Seattle Children’s Hospital, Seattle, Washington, which granted a waiver of written documentation of consent. All participants received information describing the research and indicated their consent/parental permission online.

### Measures

All data on children aged 6 to 10 years are based on parent reports. For children aged 11 to 17 years, parents were asked about family demographics and effects of COVID-19. The children in this group were asked to self-report on their physical activity, screen time, and mental health.

Demographic factors that were collected included child and parent age, child and parent sex, child and parent race and ethnicity, parents born outside the US, and parental educational attainment. Information on race and ethnicity was collected by parent report using US Census categories to account for structural racism given known disparities in the effects of COVID-19 by race and ethnicity in the US.^36^

#### Health Behaviors

Physical activity and screen time were assessed using questions from the Youth Risk Behavior Surveillance Survey (adapted for parent response for younger children),^14^ all of which have been shown to have acceptable test-retest reliability.^37^ Although no study has reported the validity of self-reported behaviors on the Youth Risk Behavior Surveillance System, a similar physical activity item has been shown to have acceptable concurrent validity with accelerometer-measured physical activity.^38^ Child physical activity was queried by asking how many days in the past week the child engaged in at least 60 min/d of physical activity (ie, activity that increased their heart rate or made them breathe hard). We categorized these responses into 3 categories: 0, 1 to 6, and 7 d/wk. This categorization allowed us to compare those not reporting physical activity of 60 min/d on any days of the week with those reporting it on some days and those meeting physical activity recommendations. The child’s daily nonacademic, recreational screen hours were calculated by adding their report of the number of hours per day spent watching television shows, movies, online videos, and video games and nonschool computer use.

#### Mental Health

Child mental health was assessed using the Strengths and Difficulties Questionnaire (SDQ), with the parent-reported version for children aged 6 to 10 years and the self-reported version for those aged 11 to 17 years. The SDQ total difficulties score (range, 0-20, with higher scores indicating greater psychopathology), which is the sum of the emotional, peer, behavioral, and hyperactivity subscales, has been found to be a psychometrically sound measure of overall child mental health problems.^39^ Per recommendation for use in general population samples, we also calculated scores for internalizing problems (emotional plus peer subscales [10 items; higher scores indicate more internalizing symptoms]) and externalizing problems (behavioral plus hyperactivity subscales [10 items; higher scores indicate more externalizing symptoms]).^40^ We did not analyze data from the peer subscale given lack of typical peer contact for many participants during the pandemic. Parents were also asked to report whether their child had ever been diagnosed or evaluated for mental or behavioral health conditions, including anxiety, depression, attention-deficit/hyperactivity disorder (ADHD), or behavioral problems. For analyses, we combined anxiety and depression as internalizing conditions and ADHD and behavioral problems as externalizing conditions. Parent mental health was assessed using the Patient Health Questionnaire for Depression and Anxiety, a validated screening measure for adult anxiety and depression.^41^

#### COVID-19 Exposure and Impact

A shortened version of the COVID-19 Exposure and Family Impact Survey was used to assess the effect of COVID-19 on the family.^42^ The COVID-19 Exposure and Family Impact Survey was developed using a rapid iterative process for ongoing and new studies in which COVID-19 may influence study outcomes. It conceptualizes exposure to potentially traumatic aspects of COVID-19 and assesses the effect of the pandemic on the family through the caregiver report. Three dimensions of this scale were used analytically: total impact (9 items), which consists of 6 items related to COVID-19 exposure (items related to family members being essential and/or health care workers or actual family exposure or illness/death related to COVID-19) and 3 items related to COVID-19 economic impact (items related to food insecurity, loss of income, and loss of health insurance).

### Statistical Analyses

Minimal data were missing because all the data were from participants who agreed to participate and were willing to answer the survey questions. For summary statistics, we reported weighted means and SDs for continuous variables and weighted frequency tables for categorical variables. Associations among mental health outcomes, COVID-19–related distress, child physical activity level, and screen hours were examined using weighted linear regression models, with all variables entered together in the model while controlling for child age, sex, race and ethnicity, parents born outside the US, parental educational attainment, school operating status, and prior diagnosis or concern for anxiety/depression or ADHD/behavioral problems as covariates. For analyses within groups aged 6 to 10 and 11 to 17 years, sampling weights were recalibrated to reflect the subsetting of the study sample. Statistical significance was set at 2-sided *P* < .05 for all analyses. All analyses were conducted using the R statistical software, version 4.0.3 (R Program for Statistical Computing).^43^

## Results

Participant characteristics are displayed in Table 1, overall and by age group. The mean (SD) age of children overall was 10.8 (3.5) years. Among the 1000 children included in the analysis, 517 (52.6%) were boys and 467 (47.4%) were girls. Two hundred ninety-three children (31.6%) in the sample were American Indian/Alaska Native, Asian, Black, or other race; 233 (27.8%) were Hispanic/Latino. In the whole sample, 220 children (22.2%) were attending school in person; 494 (50.6%), virtually; and 285 (27.2%), in a hybrid arrangement. Among the parents, 211 (24.4%) were born outside of the US and 322 (37.6%) did not complete high school.

**Table 1.** Participant Characteristics

Characteristic	Age group, y^a^		
	6-10 (n = 500)	11-17 (n = 500)	Combined (n = 1000)
**Child **			
Age, mean (SD), y	8.1 (1.4)	14.0 (2.0)	.8 (3.5)
Sex			
Male	265 (52.6)	252 (50.5)	517 (52.6)
Female	228 (47.4)	239 (49.4)	467 (47.4)
Race			
American Indian/Alaska Native	14 (2.6)	5 (1.5)	19 (2.1)
Asian	9 (2.9)	14 (2.7)	23 (2.8)
Black	59 (12.3)	52 (9.9)	111 (10.5)
White	353 (67.4)	354 (70.8)	707 (68.4)
Other^b^	65 (14.9)	75 (15.1)	140 (16.2)
Hispanic ethnicity	120 (27.6)	113 (30.7)	233 (27.8)
**Parent **			
Age, mean (SD), y	40.3 (9.0)	41.9 (8.5)	40.0 (8.2)
Sex			
Male	203 (40.6)	237 (44.1)	440 (44.6)
Female	297 (59.4)	263 (55.9)	560 (55.4)
Born outside the US	100 (22.1)	111 (26.8)	211 (24.4)
Educational attainment			
Did not complete high school	170 (38.3)	152 (36.8)	322 (37.6)
Some college	156 (27.5)	167 (26.6)	323 (26.4)
Graduated college	115 (21.8)	109 (23.1)	224 (22.5)
Postgraduate degree	59 (12.4)	72 (13.4)	131 (13.4)
Employment			
Parent(s) working full time	177 (33.5)	184 (38.1)	361 (36.1)
≥1 Parent working part time	244 (49.0)	235 (44.1)	479 (49.2)
Parent(s) unemployed	79 (17.5)	81 (17.8)	160 (14.7)

^a^ Unless otherwise indicated, data are expressed as unweighted numbers (weighted %). Mean (SD) data are weighted.

^b^ Includes Native Hawaiian or Pacific Islander and some other race if they did not identify with the categories listed.

With regard to health behaviors, children had a mean (SD) of 3.9 (2.2) d/wk with at least 60 minutes of physical activity (Table 2). Of the whole sample, 195 (20.9%) reported meeting recommendations of at least 60 minutes of physical activity every day and 90 (8.4%) reported that they had 0 days with at least 60 minutes of physical activity. Children reported a mean (SD) of 4.4 (2.5) h/d of recreational screen time.

**Table 2.** Children’s Physical Activity, Screen Time, Mental Health, and COVID-19

Measure	Age group, y^a^		
	6-10 (n = 500)	11-17 (n = 500)	Combined (n = 1000)
Physical activity			
60 min/d, mean (SD), d/wk	4.1 (2.2)	3.5 (2.1)	3.9 (2.2)
No. (%) of children, d/wk			
0	32 (7.5)	58 (11.0)	90 (8.4)
1-6	349 (69.2)	360 (75.5)	709 (70.8)
7 (recommended amount)	119 (23.3)	76 (13.5)	195 (20.9)
Unknown	0	6 (0.0)	6 (0.0)
Recreational (nonacademic) screen time, h/d	4.0 (2.4)	5.1 (2.6)	4.4 (2.5)
Mental health			
Child total SDQ score^b^	12.1 (7.1)	11.7 (7.36)	11.9 (7.1)
Child SDQ externalizing score^c^	6.8 (4.3)	5.8 (4.1)	6.3 (4.1)
Child SDQ internalizing score^d^	5.3 (3.7)	5.8 (4.0)	5.6 (3.8)
COVID-19 stressors			
Total impact score^e^	2.4 (1.8)	2.5 (1.8)	2.4 (1.8)
Exposure score^f^	1.5 (1.3)	1.6 (1.4)	1.6 (1.4)
Economic impact^g^	0.9 (0.9)	0.9 (1.0)	0.9 (0.9)
Prior conditions diagnosed or under evaluation, No. (%)			
Anxiety	47 (9.2)	96 (19.7)	143 (13.7)
Depression	24 (5.2)	86 (19.7)	110 (10.4)
ADHD	72 (13.9)	88 (18.4)	160 (15.0)
Behavioral problem	59 (12.3)	57 (13.2)	116 (11.4)
Current school status, No. (%)			
In person	124 (24.7)	96 (17.1)	220 (22.2)
Hybrid (in person plus remote)	132 (24.7)	153 (32.4)	285 (27.2)
Remote/virtual school	243 (50.6)	251 (50.6)	494 (50.6)

Abbreviations: ADHD, attention-deficit/hyperactivity disorder; SDQ, Strengths and Difficulties Questionnaire.

^a^ Unless otherwise indicated, data are expressed as weighted mean (SD). Numbers are nonweighted; percentages are weighted.

^b^ Scores range from 0 to 20, with higher scores indicating greater psychopathology.

^c^ Scores range from 0 to 10, with higher scores indicating more externalizing symptoms.

^d^ Scores range from 0 to 10, with higher scores indicating more internalizing symptoms.

^e^ Scores range from 0 to 9, with higher scores indicating greater impact of COVID-19 pandemic on the family.

^f^ Scores range from 0 to 6, with higher scores indicating greater exposure of the family to COVID-19 pandemic–related events.

^g^ Scores range from 0 to 3, with higher scores indicating a greater economic impact of the COVID-19 pandemic on the family.

Overall, 143 children (13.7%) were diagnosed with or were undergoing evaluation for anxiety; 110 (10.4%), depression; 160 (15.0%), ADHD; and 116 (11.4%), a behavioral problem. The mean (SD) SDQ score for total difficulties was 11.9 (7.1). Additional details, including results from the SDQ, are reported in Table 2.

Results from the regression analyses are shown in Table 3, with all models adjusted for child age, sex, race and ethnicity, parental educational attainment, parent born outside the US, school status, and parent Patient Health Questionnaire for Depression and Anxiety score. COVID-19 was statistically significantly associated with higher total difficulties (β coefficient, 0.6; 95% CI, 0.3-0.9) and externalizing (β coefficient, 0.3; 95% CI, 0.1-0.5) and internalizing (β coefficient, 0.3; 95% CI, 0.2-0.5) symptoms for the younger group and with higher total difficulties (β coefficient, 0.4; 95% CI, 0.0-0.7) and externalizing symptoms (β coefficient, 0.3; 95% CI, 0.1-0.5) scores for the older group. For younger children, engaging in the recommended 7 d/wk of physical activity was associated with lower total difficulties (β coefficient, −2.4; 95% CI, −4.6 to −0.2) and externalizing symptoms (β coefficient, −2.0; 95% CI, −3.4 to −0.6) scores compared with those with 0 d/wk but not for internalizing symptoms. For this age group, 1 to 6 d/wk (vs 0) with at least 60 minutes of physical activity was not significantly associated with improved mental health outcomes. For older children, engaging in 1 to 6 and 7 d/wk (vs 0) of physical activity was significantly associated with lower total difficulties (β coefficients, −3.5 [95% CI, −5.3 to −1.8] and −3.6 [95% CI, −5.8 to −1.4], respectively) and externalizing (β coefficients, −1.5 [95% CI, −2.5 to −0.4] and −1.3 [95% CI, −2.6 to 0.0], respectively) and internalizing (β coefficients, −2.1 [95% CI, −3.0 to −1.1] and −2.3 [95% CI, −3.5 to −1.1], respectively) symptoms scores. Among younger and older groups, more screen time was also positively correlated with higher SDQ total difficulties (β coefficients, 0.3 [95% CI, 0.1-0.5] and 0.4 [95% CI, 0.2-0.6], respectively) and externalizing (β coefficients, 0.1 [95% CI, 0.0-0.3] and 0.2 [95% CI, 0.1-0.4], respectively) and internalizing (β coefficients, 0.1 [95% CI, 0.0-0.3] for both) symptoms scores. There were no statistically significant differences by sex in any of these models.

**Table 3.** Children’s Mental Health by COVID-19 Impact, Physical Activity, and Screen Time in a Weighted Multivariable Model^a^

Measure	SDQ total difficulties		SDQ symptoms			
			Externalizing		Internalizing	
	β (95% CI)	*P* value	β (95% CI)	*P* value	β (95% CI)	*P* value
**Children aged 6-10 y**						
COVID-19 total impact score^b^	0.6 (0.3 to 0.9)	<.001	0.3 (0.1 to 0.5)	.006	0.3 (0.2 to 0.5)	<.001
Physical activity, d/wk						
0	1 [Reference]	NA	1 [Reference]	NA	1 [Reference]	NA
1-6	−0.8 (−2.7 to 1.2)	.44	−0.9 (−2.2 to 0.3)	.15	0.2 (−0.9 to 1.2)	.76
7	−2.4 (−4.6 to −0.2)	.04	−2.0 (−3.4 to −0.6)	.006	−0.4 (−1.6 to 0.8)	.53
Screen time	0.3 (0.1 to 0.5)	.01	0.1 (0.0 to 0.3)	.05	0.1 (0.0 to 0.3)	.02
Prior anxiety and/or depression						
None	1 [Reference]	NA	1 [Reference]	NA	1 [Reference]	NA
1 Condition	−0.8 (−2.9 to 1.4)	.50	−0.9 (−2.3 to 0.5)	.19	0.2 (−1.0 to 1.4)	.75
Both conditions	−0.3 (−3.2 to 2.6)	.85	−1.0 (−2.8 to 0.9)	.31	0.7 (−0.9 to 2.2)	.4
Prior ADHD and/or behavioral problem						
None	1 [Reference]	NA	1 [Reference]	NA	1 [Reference]	NA
1 Condition	5.6 (3.7 to 7.5)	<.001	4.0 (2.8 to 5.2)	<.001	1.6 (0.6 to 2.6)	.003
Both conditions	7.2 (5.0 to 9.3)	<.001	4.4 (3.0 to 5.8)	<.001	2.8 (1.6 to 3.9)	<.001
**Children aged 11-17 y**						
COVID-19 total impact score^b^	0.4 (0.0 to 0.7)	.03	0.3 (0.1 to 0.5)	.01	0.1 (−0.1 to 0.3)	.25
Physical activity, d/wk						
0	1 [Reference]	NA	1 [Reference]	NA	1 [Reference]	NA
1-6	−3.5 (−5.3 to −1.8)	<.001	−1.5 (−2.5 to −0.4)	.006	−2.1 (−3.0 to −1.1)	<.001
7	−3.6 (−5.8 to −1.4)	.002	−1.3 (−2.6 to −0.0)	.04	−2.3 (−3.5 to −1.1)	<.001
Screen time	0.4 (0.2 to 0.6)	<.001	0.2 (0.1 to 0.4)	<.001	0.1 (0.0 to 0.3)	.01
Prior anxiety and/or depression						
None	1 [Reference]	NA	1 [Reference]	NA	1 [Reference]	NA
1 Condition	3.5 (2.0 to 5.1)	<.001	1.1 (0.2 to 2.0)	.02	2.5 (1.6 to 3.3)	<.001
Both conditions	4.8 (2.9 to 6.7)	<.001	0.9 (−0.2 to 2.0)	.12	3.8 (2.8 to 4.9)	<.001
Prior ADHD and/or behavioral problem						
None	1 [Reference]	NA	1 [Reference]	NA	1 [Reference]	NA
1 Condition	1.9 (0.3 to 3.6)	.02	1.8 (0.8 to 2.8)	<.001	0.1 (−0.8 to 1.0)	.78
Both conditions	3.1 (1.1 to 5.1)	.003	3.0 (1.8 to 4.2)	<.001	0.01 (−1.1 to 1.1)	.99

Abbreviations: ADHD, attention-deficit/hyperactivity disorder; NA, not applicable; SDQ, Strengths and Difficulties Questionnaire.

^a^ All models adjusted for child age, sex, race and ethnicity, parent educational attainment, parent born outside the US, school status, and parent Patient Health Questionnaire for Depression and Anxiety scores.

^b^ Scores range from 0 to 9, with higher scores indicating greater impact of the COVID-19 pandemic on the family.

## Discussion

Using a nationally representative US sample, we found that school-aged children are engaging in suboptimal amounts of physical activity and screen time during the COVID-19 pandemic. Notably, children who were exposed to more pandemic-related stressors engaged in less physical activity and had more screen use than their peers who were less exposed. Better health behaviors, in turn, were associated with better mental health, even when accounting for differences in exposure to pandemic-related stressors and demographic factors. These findings suggest that physical activity and screen time constitute potential health promotion targets to mitigate the effect of the pandemic on child mental health and well-being.

This study also found an association between children’s mental health and their exposure to pandemic-related stressors. The results of the SDQ scores herein were higher than previously published US norms; 1 previous national study^44^ reported mean (SD) scores for children aged 4 to 17 years of 7.1 (5.7) for total difficulties compared with 11.9 (7.1) in this sample. As expected, children whose families experienced the most pandemic-related stressors exhibited the most mental health and behavioral problems. Notably, this association persisted when controlling for demographic characteristics previously associated with health disparities and disproportionate COVID-19 impact, such as race and ethnicity, and parental educational attainment.^45^ Furthermore, parental mental health was strongly associated with child mental health, yet the impact of COVID-19 remained strongly associated with child mental health even after controlling for parental mental health.

For elementary school–aged children, our findings highlight the potential benefits of engaging in the recommended 60 minutes or more of physical activity every day. Even after accounting for COVID-19 stressors and demographics, achieving that recommendation was associated with fewer externalizing symptoms. Given that fewer than one-quarter of children aged 6 to 10 years in our sample were meeting physical activity recommendations, universal efforts to increase child physical activity have substantial potential to improve population health and well-being. For this age group, particularly if they are not attending school in person, the responsibility of providing opportunities for physical activity falls on parents and caregivers, which may pose considerable challenges. Furthermore, there may be disparities in access to opportunities to engage in physical activity, whether indoors (space, online access, and equipment) or outdoors (space, safety concerns, and equipment). Equitably increasing opportunities for physical activity would require schools to play a central role so that access does not depend on family or neighborhood circumstances. Recess and physical education during the school day (in person or remotely) need to be prioritized and creative solutions considered.

For middle and high school–aged children, the results for physical activity were more striking. Only 13.5% of these children are engaging in the recommended 60 minutes of physical activity daily, which is considerably lower than prepandemic estimates of approximately 25%.^4^ Notably, even 1 d/wk of physical activity was associated with better mental health than 0 days for both externalizing and internalizing symptoms. This significant association remained even when accounting for prior mental health conditions, COVID-19 stressors, and demographics. Unfortunately, physical activity among US children tends to decline as they get older and often depends on participation in sports and organized physical activities,^46^ which were severely curtailed during the pandemic. As with younger children, school-based options should be supported, along with collaboration between schools and community-based organizations to increase opportunities for affordable and accessible after-school recreation and sports. For this age group, it will be important to monitor possible attrition from organized physical activities after the pandemic owing to the long periods of restrictions.

In both age groups, more screen time was associated with worse mental health. This finding is consistent with those from prepandemic studies^21,22^ but is especially problematic during this time of prolonged restrictions on typical school, extracurricular, and social opportunities. For children engaging in remote or hybrid schooling, there are likely many additional hours of academic screen time. Of note, our estimates for daily recreational screen time are in line with prepandemic estimates that range from 4 to more than 7 h/d^19,20^ but are difficult to compare given the granularities of the measures. The Kaiser Family Foundation’s estimate of 7.5 h/d of recreational media use is based on a more extensive survey that also includes music and audio time and multitasking questions. Although our survey question focused on nonacademic screen hours, it is possible that multitasking on screens (such as telephone use during class) during remote school time was underestimated.^20^ Although a direct comparison with prepandemic estimates of screen time may not be possible, the association between more screen time and worse mental health was clear. Although recognizing that some prepandemic screen time rules may need to be modified, parents and teachers should encourage non–screen-based activities whenever possible. In addition, a harm reduction approach may be appropriate so that even if screen time is higher than ideal, engaging in physical activity may offer independent benefits to health and well-being.

### Strengths and Limitations

The strengths of this study include its large sample size with nationally comparable demographics and rigorous survey study methods. Its limitations include the fact that all data were collected by parent or child report and are subject to recall and social desirability biases. We attempted to mitigate biases due to parent report by asking older children directly about their health behaviors and mental health. We also used validated, reliable measures whenever available. We were unable to discern whether mental health diagnoses and concerns started before or during the pandemic, and this factor could be the reason that rates of mental health concerns and diagnoses are higher in our sample than in those of other reports.^47^ Because this was a cross-sectional study, we also could not assess whether health behaviors changed during the pandemic or infer causality. Children who engage in less physical activity may experience more mental health symptoms, or children who have more mental health symptoms may have difficulty attaining the recommended amount of activity. Longitudinal or intervention studies during the pandemic would be needed to examine causality; however, previous literature on both physical activity and screen time^5,7,21,22^ provides evidence that a causal relationship exists between the health behaviors and mental health. Finally, this survey was conducted during the fall of 2020, and pandemic-related circumstances—including COVID-19 rates, state mandates and restrictions, and the status of schools, sports, and park access—were variable across the country and may have changed in the months that followed.

## Conclusions

Although much of the immediate health concerns related to the COVID-19 pandemic have focused on infection and its sequelae, this cross-sectional study highlights the critical need to also address the short- and long-term consequences of the pandemic on the mental health of children. Although there is notable concern regarding pandemic-related increases in weight gain and obesity rates,^48,49^ this study underscores that the same behaviors of screen time and physical inactivity are also associated with poorer mental health outcomes in children. Before the pandemic, three-quarters of school-aged children in the US failed to meet physical activity guidelines; this situation has been exacerbated by pandemic-related circumstances and is particularly worse for middle and high school students. There is thus a critical need to reimagine multisector approaches to providing equitable opportunities for physical activity, including sports and outdoor recreation, for all children.
